# TreeKernel: interpretable kernel machine tests for interactions between -omics and clinical predictors with applications to metabolomics and COPD phenotypes

**DOI:** 10.1186/s12859-023-05459-x

**Published:** 2023-10-25

**Authors:** Charlie M. Carpenter, Lucas Gillenwater, Russell Bowler, Katerina Kechris, Debashis Ghosh

**Affiliations:** 1 Department of Biostatistics and Informatics, University of Colorado Denver, Anschutz Medical Campus, Denver, CO USA; 2 Computational Bioscience Program, University of Colorado Denver, Anschutz Medical Campus, Denver, CO USA; 3 Department of Medicine, National Jewish Health, Denver, USA; 4 University of Colorado Denver, Anschutz Medical Campus, Denver, CO USA

**Keywords:** Kernel functions, Kernel methods, Kernel interactions, Metabolomics, Metabolic pathways

## Abstract

**Background:**

In this paper, we are interested in interactions between a high-dimensional -omics dataset and clinical covariates. The goal is to evaluate the relationship between a phenotype of interest and a high-dimensional omics pathway, where the effect of the omics data depends on subjects’ clinical covariates (age, sex, smoking status, etc.). For instance, metabolic pathways can vary greatly between sexes which may also change the relationship between certain metabolic pathways and a clinical phenotype of interest. We propose partitioning the clinical covariate space and performing a kernel association test within those partitions. To illustrate this idea, we focus on hierarchical partitions of the clinical covariate space and kernel tests on metabolic pathways.

**Results:**

We see that our proposed method outperforms competing methods in most simulation scenarios. It can identify different relationships among clinical groups with higher power in most scenarios while maintaining a proper Type I error rate. The simulation studies also show a robustness to the grouping structure within the clinical space. We also apply the method to the COPDGene study and find several clinically meaningful interactions between metabolic pathways, the clinical space, and lung function.

**Conclusion:**

TreeKernel provides a simple and interpretable process for testing for relationships between high-dimensional omics data and clinical outcomes in the presence of interactions within clinical cohorts. The method is broadly applicable to many studies.

**Supplementary Information:**

The online version contains supplementary material available at 10.1186/s12859-023-05459-x.

## Background

In this paper, we are interested in interactions between a high-dimensional -omics dataset and clinical covariates. The goal is to evaluate the relationship between a phenotype of interest and a high-dimensional omics pathway, where the effect of the omics data depends on subjects’ clinical covariates (age, sex, smoking status, etc.). For instance, metabolic pathways can vary greatly between sexes [1–3] which may also change the relationship between certain metabolic pathways and a clinical phenotype of interest, e.g. body compositions.

One common way of testing for relationships between omics pathways and phenotypes is to represent omics data with a kernel machine [4, 5]. These kernel association tests test for relationships between the clinical phenotype of interest and an entire omics profile [6] or important subsets of omic features [7–9]. These methods all model a constant relationship between the outcome space and the kernel space after controlling for clinical covariates. There has been a large effort to extend these methods for multimodal data sets [10–12]. The goal of these studies is to integrate multiple high-dimensional data sets to better understand intertwined biological systems. These methods are designed to model interactions between the omics features. Other kernel interaction techniques look for feature-to-feature interactions within the same set [13]. While powerful, these methods do not provide easily interpretable interactions and are not built for interactions between clinical and omics features.

We propose partitioning the clinical covariate space and performing a kernel association test within those partitions. There are many ways to partition spaces. One common method is $kd$-trees [14], where $k$ represents the number of variables in the space and $d$ is the depth of the tree. In the simplest model, one partitions based on the median of each variable sequentially. More complex algorithms that consider all $k$ variables at once or use measures other than the median may be used as well. The hierarchical clustering algorithm [15] is another classic algorithm that can be used to create partitions in the data. This algorithm results in a dendrogram that can then be “cut” at different heights, resulting in different partitions of the data.

Testing on hierarchical structures has been studied by many authors. Some authors group individual analytes (genes, microbes, etc.) into trees and test for a relationship with each analyte [16]. We are more interested in grouping subjects and testing within those subgroups. Yekutieli studied controlling the false discovery rate for multiple hypothesis tests with a hierarchical testing structure [17]. Bogomolov et al. [18] also considered this setting and added the concept of tests being nested within one another. This nesting was represented using a tree structure, and the resulting procedure led improved power over Yekutieli’s [17] approach. Multiscale test corrections [19, 20] are another method for controlling the error rate from multiple structured tests and have been studied under a hierarchical setting [21]. Some hierarchical kernel tests have also been developed [22]. These do not consider new relationships within covariate partitions, but rather a hierarchy of importance of omics pathways.

We propose a new approach to produce interpretable interactions between clinical covariate spaces and kernel spaces. First, we partition the covariate space into a hierarchical structure; second, we perform kernel association tests between the outcome and the subjects within each partition. The former step involves the clinical covariates, while the latter step tests for association between the omics data and the outcome. This simple test construction, which we call *TreeKernel*, provides interpretable interactions between omics data and clinical covariates. We explore the validity of this approach through simulations and analysis of a metabolomics data set. We find that we achieve good power in detecting interactions between simulated clinical covariate and metabolic pathways and that the nominal Type I error rate is preserved. Analysis of the metabolomics pathways show that the relationship between lung capacity and certain metabolic pathways vary depending on the patients’ smoking status.

## Methods

### Kernel and covariate spaces

This paper is primarily concerned with interactions between observed clinical covariates and omics data. We frame this as an interaction between the clinical covariate space and the omics space. Kernel functions map data from a high dimensional observation space, ${\mathcal{Z}},$ to a feature space using a nonlinear feature map. For this work, we refer to a kernel function as any bivariate symmetric function $h\left( {x,z} \right)$ on ${\mathcal{Z}} \times {\mathcal{Z}}$ for which $\mathop \smallint \limits_{{\mathcal{Z}}} \mathop \smallint \limits_{{\mathcal{Z}}} h\left( {x,z} \right)g\left( x \right)g\left( z \right)dxdz \ge 0$ for all squared integrable functions $g$ on ${\mathcal{Z}}$, i.e., $g \in L^{2} \left( {\mathcal{Z}} \right)$. It is known that for every positive definite kernel $h$, there exists a unique Hilbert space, ${\mathcal{H}}$, of functions on ${\mathcal{Z}}$ for which the function value is reproduced by the kernel, known as the reproducing kernel Hilbert space (RKHS) [23]. The RKHS formulation implies that a given function, $f \in {\mathcal{H}}$ on ${\mathcal{Z}}$, can be expressed as $f\left( Z \right) = \left\langle {f\left( \cdot \right), h\left( { \cdot , Z)} \right.} \right\rangle_{{_{{\mathcal{H}}} }}$, where $\left\langle { \cdot , \cdot } \right\rangle_{{_{{\mathcal{H}}} }}$ is the inner product of ${\mathcal{H}}$ and $Z \in {\mathcal{Z}}$ is an observed point. One may define a nonlinear (or linear) feature map ${{\varvec{\Phi}}}: {\mathcal{Z}} \to {\mathcal{H}}$ as $\phi \left( Z \right) = h\left( { \cdot , Z} \right).$ Replacing $f$ with $h\left( { \cdot , \tilde{Z}} \right)$ gives $h\left( {Z, \tilde{Z}} \right) = \left\langle {h\left( { \cdot , Z} \right),h\left( { \cdot ,\tilde{Z}} \right)} \right\rangle_{{\mathcal{H}}}$, and, finally, the famous “kernel trick” gives $h\left( {Z,\tilde{Z}} \right) = \left\langle {\phi \left( Z \right),\phi \left( {\tilde{Z}} \right)} \right\rangle_{{\mathcal{H}}}$, [24, 25]. In words, the kernel function represents the inner product between two vectors within the feature space efficiently without needing to explicitly define the form of the feature map, $\phi \left( \cdot \right)$., or the space, ${\mathcal{H}}$.

### Kernel association tests

We will assume that omics data are properly normalized and contain no missing values. Consider a dataset with $n$ observations. Let $Y$ be a vector of length $n$ representing a continuous or discrete outcome. Let $C$ be an $n \times q$ matrix of clinical covariates and $Z$ be an $n \times m$ matrix of high-dimensional biological data. The classic semi-parametric kernel machine model [1, 2] then relates these three through the model

1 $$g\left( Y \right) = {\text{C}}\beta + h\left( Z \right) + \epsilon ,$$

where $g$ is either the identity or *logit* link function, $\beta$ is a $q \times 1$ vector of regression coefficients, $\epsilon$ is an $n \times 1$ vector of error terms, and $h$ is a kernel function.

The kernel function, $h$, can be considered a measure of similarity between two subjects within the kernel space. Some common kernel machine representations for $h$ include the Linear Kernel: $K\left( {z_{i} ,z_{j} } \right) = z_{i}^{T} z_{j}$ (the dot product), the dth Polynomial Kernel: $K\left( {z_{i} ,z_{j} ,\rho } \right) = \left( {z_{i}^{T} z_{j} + \rho } \right)^{d}$, and the Gaussian Kernel: $K\left( {z_{i} ,z_{j} ,\rho } \right) = \exp \left\{ { - \frac{{\parallel z_{i} - z_{j}\parallel _2^{2} }}{\rho }} \right\}{ }$, where $\parallel \cdot \parallel_2$ is the Euclidean ($L_{2}$) norm. For the Gaussian kernel, $\rho$ is a precision parameter controlling how quickly similarities approach 0. We will use the median of all pairwise Euclidean distances from $Z$ as an empirical estimate of $\rho$ in our Gaussian kernels.

### Proposed method

We first represent the clinical covariates using a lower-dimensional space that captures their underlying variation. This can be accomplished by embedding the data using their principal components if all data are continuous. If the data contain both continuous and factor variables, the primary left singular vectors from the factor analysis of mixed data are used as covariates [26]. The partitions calculated on this embedding will be ignoring the raw noise in the clinical space encoded in the removed left singular vectors. We then cluster the data within this embedding to create data partitions. Many clustering methods may be appropriate, e.g., k-means or kd-trees. We use hierarchical clustering for TreeKernel as we find improvements in power using tree-based testing corrections. The number of clusters are estimated using the highest relative loss of inertia. Partitions are derived from the clusters calculated from each tree cut, and we assume that these partitions give reasonably homogeneous grouping of clinical factors.

**Fig. 1:**
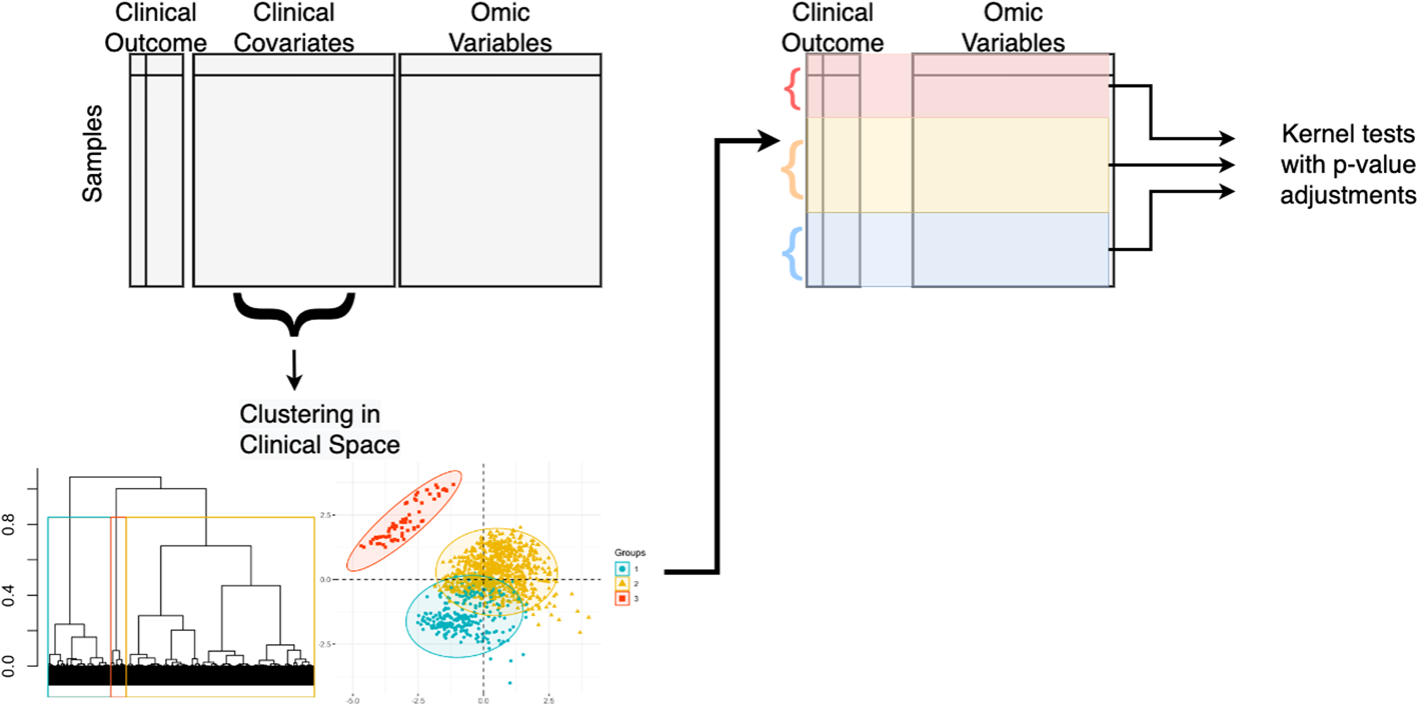
Flowchart of the TreeKernel workflow. Subjects are clustered based on their clinical data, then kernel association tests are performed within these partitions with p-values adjustments applied at the end. This allows for interpretable interactions between omics and clinical variables

Once the $p$ partitions are identified, we perform kernel association tests between the outcome of interest and the kernel space within the partitions. I.e., we perform a kernel association test using the model

2 $$g\left( {Y_{i}^{\left( p \right)} } \right) = ({\text{C}}\beta )^{\left( p \right)} + h\left( {Z_{i}^{\left( p \right)} } \right) + \epsilon_{i}^{\left( p \right)} ,$$

where $\beta_{0}^{\left( p \right)}$ is the intercept within partition $p$ and $Y^{\left( p \right)} ,({\text{C}}\beta )^{\left( p \right)} , Z^{\left( p \right)} ,$ and $\epsilon^{\left( p \right)}$ are the outcome, clinical covariates, high dimensional biological data, and model residuals from within each partition, respectively. Each model is fit separately. Finally, we perform the multiple testing correction procedure TreeBH [18] which controls the global error rates on hypotheses that are organized hierarchically in a tree structure. Our workflow is visualized in Fig. 1.

### Simulation study

We conducted multiple simulation studies to assess the power and nominal Type I error rates of our proposed method using R [27]. We first simulated our $n \times m$ dimensional set, $Z$, to mimic metabolic abundance within connected pathways. Random graphs were generated from the *igraph* package in R [28] and $Z$ was generated using the same method described in [9]. We Then simulated a clinical covariate space, $C$, with four variables and 2, 3, or 4, distinct partitions. We refer to these settings as “4-partition,”, “3-partition,”, and “2-partition.” First, we simulated data from a multivariate normal distribution, $MVN\left( {\mu , I} \right)$, where $I$ is the identity matrix. The mean vector, ${\varvec{\mu}}$, is a constant vector of one of **0**, **2**, **4**, or **8** for the different partitions. For example, the 2-partition simulations draw half of the total sample size from the $MVN$ distribution with a vector mean of **0** and the other half from a distribution with a mean vector of **2**. Next, we simulated data sets with categorical variables driving the clustering. Three uniform distributed variables with a range of (− 0.15, 0.15), (− 1, 1), and (0, 4), respectively, were simulated. We then generated a fourth *partitioning* variable from a factor variable with 4, 3, or 2 levels for the partition settings. The final *partitioning* variable came from an interaction between two factor variables with two levels. This gives only a 4-partition scenario. All simulations settings had a sample size of 200 and were repeated 2,000 times. We simulate 50 observations within each partition in the 4-partition setting. In the 3-partition setting, we simulate 66, 66, and 68 observations per partition, respectively. Finally, we simulate 100 observations per partition in the 2-partition setting.

This covariate space was then embedded using their principal components or left singular vectors from factor analysis and partitioned with hierarchical clustering. Within each partition, an outcome was simulated as $Y_{i} = C_{i} \beta + h_{p} \left( {Z_{i} } \right) + \epsilon_{i}$, where $\beta$ is the vector (1, 0.05, − 0.26, 0.1, − 0.1, 0.26, − 0.02), which come from observed relationships in our metabolomics cohort, and $\epsilon_{i}$ is a normally distributed random variable with mean 0 and standard deviation 1.3688. This standard deviation was also drawn from observed metabolomics data. We used a linear kernel in all settings, i.e., $h_{p} \left( {Z_{i} } \right) = b_{p} \cdot Z_{i}$. For power calculations in the 4-partition and 3-partition, we had two sub-settings with either 1 active group, $b_{1} = 0.5$ and $b_{2} = b_{3} = b_{4} = 0$, or two active groups, $b_{1} = b_{2} = 0.3,$ and $b_{3} = b_{4} = 0.$ For the power calculations in the 2-partition, we had only had one setting, $b_{1} = 0.5$ and $b_{2} = 0.$ For the Type I error rate estimation all $b_{p} = 0.$ We repeated these simulations with three pathway sizes, $m$ = 15, 30, or 45. We performed each of these simulations using either 3 or 5 embedding components for clustering to assess the sensitivity. Lastly, we compare the power of our method to two simple competing approaches: an F-test on all principal components (FPC) of ${\varvec{Z}}$ [29] and the minimum Simes’ adjusted *p*-value [30] from univariate tests on ${\varvec{Z}}$ (Univariate Simes). The code for all our simulations can be found at https://github.com/Ghoshlab/TreeKernel.

### COPDGene data

We analyzed data collected from the COPDGene study [31], a multicenter observational study that collected genetic data as well as multiple measures of lung function to study chronic obstructive pulmonary disease (COPD). Between 2007 and 2011, 10,198 participants with and without COPD enrolled (Visit 1). A 5-year follow up visit took place between 2013 and 2017 (Visit 2). Blood samples were also obtained for -omics analyses from participants who provided consent. In total, 1136 subjects (1040 non-Hispanic white, 96 African American) participated in a metabolomics ancillary study in which they provide fresh frozen plasma collected using an 8.5 mL p100 tube (Becton Dickson) at Visit 2.

### Metabolomics and data processing

P100 plasma was profiled using the Metabolon (Durham, NC, USA) Global Metabolomics platform. Briefly, untargeted liquid chromatography–tandem mass spectrometry (LC–MS/MS) was used to quantify 1392 metabolites and described in [32, 33]. A data normalization step was performed to correct variation resulting from instrument inter-day tuning differences: metabolite intensities were divided by the metabolite run day median, then multiplied by the overall metabolite median. It was determined that no further normalization was necessary based on the reduction in the significance of association between the top PCs and sample run day after normalization. Subjects with aggregate metabolite median *z*-scores greater than 3.5 standard deviation from the mean (*n* = 6) of the cohort were removed. Metabolites were excluded if > 20% of samples were missing values [34]. For the 995 remaining metabolites, missing values were imputed across metabolites with k-nearest neighbors imputation (*k* = 10) using the R package *impute* [35]. As a final step, metabolomic data was log transformed and standardized. Linear regression models were fit to each metabolite controlling for white blood cell count, percent eosinophil, percent lymphocytes, percent monocytes, percent neutrophils, and hemoglobin. The partial residuals were then used as the observed metabolomics data. These data are available at Metabolomics Workbench [36] with identifier PR000907.

Four hundred and thirty six of these metabolites had an id in the KEGG database of human pathways, which was accessed using the *keggLink* function from the *KEGGREST* package [37]. These 436 metabolites appear in 161 KEGG pathways, and 29 of these 161 KEGG pathways contained 10 or more metabolites. This cutoff was to ensure that our observed pathways aren’t too small. Note that our filtered dataset did not contain every metabolite within the 29 KEGG pathways selected, and therefore some of the analyzed pathways have only 10 metabolites. Edges in a pathway’s graph were defined by connections within a pathway from the KEGG reaction database.

### Analysis

We focus on two COPD phenotypes: (1) percent emphysema and (2) the ratio of post-bronchodilator forced expiratory volume at one second divided by forced vital capacity (FEV_1_/FVC). Emphysema, a measure of erosion of the distal airspaces, has been linked with the clinical severity of COPD [38]. It is an imaging-based phenotype defined as the 15th percentile lung voxel density in Hounsfield units adjusted for total lung capacity from quantitative CT imaging analyses. FEV_1_/FVC is a measure of airflow obstruction. To normalize FEV_1_/FVC, we use the following log ratio transformation, ${\text{log}}\left( {\left( {\frac{{FEV_{1} }}{FVC}} \right) /\left( {1 - \frac{{FEV_{1} }}{FVC}} \right)} \right)$. After removing incomplete cases we were left with 1,113 complete cases for the FEV_1_/FVC analysis and 1,065 complete cases for the percent emphysema analysis.

Our clinical covariates were age, sex, BMI, smoking pack years, clinical center, and smoking status (current, former, never). We performed a factor analysis of mixed data on these clinical covariates and hierarchical clustering on the first 5 left singular vectors using the *FactoMineR* package in R [39]. We then used PaIRKAT [9] to test for relationships between the outcomes and the selected metabolic pathways within the partitions and applied the TreeBH correction to p-values. In our analysis, the patients grouped into 1 large group (former smokers, n $\approx$ 785) and 2 smaller groups (current n $\approx$ 260, and never smokers n $\approx$ 65). Many pathways were significant associated with the outcomes in the overall group and former smokers but not the other groups. We believe this had most to do with the differences in sample size, so we randomly downsampled the former smoking group to n = 275 and performed the test on this subset. We repeated this 100 times and reported the average p-values. The current and never smokers were assessed using all subjects in those groups. We do not recommend this in a formal analysis. We only do this as a sensitivity analysis of our method.

## Results

### Simulation results

The estimated power from the simulation using 1 Normal partition variable and 5 components for clustering are displayed in Table 1. The Univariate Simes approach had lower power than TreeKernel in almost every setting. We this method have the highest power in detecting the two active groups with 4 partitions in simulated omics sets of only 15 variables. The FPC test has the highest power when there is only one active partition in the 4-partition setting with 15 omics variables. With fewer partitions or larger simulated omics pathways we see that TreeKernel has the highest power in every setting. This general patter repeats for all simulation studies. The FPC test has the highest power in the smallest simulated omics pathways with 4 partitions, and TreeKernel has the best power in all other settings whether it is one factor variable (Table 2) or two factor variables (Table 3) creating the partitions. The pattern is also there when we only 3 PCs or left singular vectors for clustering (Additional file 1: Tables S1–S3).

**Table 1:** Estimated power from 2000 simulations from a multivariate normal distribution with 2, 3, or 4 partitions with 15, 30, and 45 omics variables

	Test					
	TreeKernel		Univariate Simes		Principal Component F-test	
*15 omics variables*						
4-partition						
2 Active groups	Group 1	Group 2	Group 1	Group 2	Group 1	Group 2
	0.563	0.214	**0.673**	**0.334**	0.154	0.107
1 Active group	0.740	0.696	**0.832**			
3-Partition						
2 Active groups	Group 1	Group 2	Group 1	Group 2	*Group 1*	*Group 2*
	**0.898**	**0.613**	0.597	0.023	0.833	0.018
1 Active group	**0.978**	0.843	0.918			
2-Partition						
1 Active group	**0.998**	0.965	0.962			
*30 omics variables*						
4-Partition						
2 Active groups	Group 1	Group 2	Group 1	Group 2	*Group 1*	*Group 2*
	**0.633**	**0.338**	0.298	0.099	0.443	0.326
1 Active group	**0.723**	0.510	0.621			
3-Partition						
2 Active groups	Group 1	Group 2	Group 1	Group 2	*Group 1*	*Group 2*
	**0.909**	**0.771**	0.486	0.107	0.656	0.020
1 Active group	**0.980**	0.703	0.766			
2-Partition						
1 Active group	0.998	0.931	0.884			
*45 omics variables*						
4-Partition						
2 Active groups	Group 1	Group 2	Group 1	Group 2	*Group 1*	*Group 2*
	**0.706**	**0.691**	0.219	0.109	0.359	0318
1 Active group	**0.704**	0.358	0.508			
3-Partition						
2 Active groups	Group 1	Group 2	Group 1	Group 2	*Group 1*	*Group 2*
	**0.912**	**0.881**	0.347	0.017	0.551	0.016
1 Active group	**0.960**	0.556	0.659			
2-Partition						
1 active group	**0.996**	0.885	0.799			

Tests used five principal components for clustering. Bold cells indicate the top performance within the simulation. ‘Group 1’ and ‘Group 2’ refer to the two partitions where the outcome was related to the simulated pathway

**Table 2:** Estimated power from 2000 simulations with 15, 30, and 45 omics variables using five components for clustering with 1 categorical grouping variable

	Test					
	TreeKernel		Univariate simes		Principal component F-test	
*15 omics variables*						
4-Partition						
2 Active groups	Group 1	Group 2	Group 1	Group 2	Group 1	Group 2
	0.657	0.323	0.011	0.008	**0.731**	**0.413**
1 Active group	0.836	0.033	**0.869**			
3-Partition						
2 Active groups	Group 1	Group 2	Group 1	Group 2	Group 1	Group 2
	**0.895**	**0.586**	0.011	0.014	0.841	0.015
1 Active group	**0.985**	0.036	0.924			
2-Partition						
1 Active group	**0.998**	0.038	0.973			
*30 omics variables*						
4-Partition						
2 Active groups	Group 1	Group 2	Group 1	Group 2	Group 1	Group 2
	**0.673**	**0.461**	0.008	0.008	0.542	0.379
1 Active group	**0.825**	0.036	0.682			
3-Partition						
2 Active groups	*Group 1*	*Group 2*	*Group 1*	*Group 2*	*Group 1*	*Group 2*
	**0.923**	**0.774**	0.010	0.013	0.658	0.018
1 Active group	**0.982**	0.029	0.759			
2-Partition						
1 Active group	**0.989**	0.036	0.880			
*45 omics variables*						
4-Partition						
2 Active groups	Group 1	Group 2	Group 1	Group 2	Group 1	Group 2
	**0.715**	**0.583**	0.008	0.007	0.429	0.373
1 Active group	**0.759**	0.030	0.537			
3-Partition						
2 Active groups	Group 1	Group 2	Group 1	Group 2	Group 1	Group 2
	**0.928**	**0.881**	0.011	0.012	0.559	0.024
1 Active group	**0.967**	0.036	0.666			
2-Partition						
1 Active group	**0.997**	0.033	0.792			

‘Group 1’ and ‘Group 2’ refer to the two partitions where the outcome was related to the simulated pathway

Bold cells indicate highest power in the simulation setting

**Table 3:** Estimated power from 2000 simulations with 15, 30, and 45 omics variables using five components for clustering with 2 discrete grouping variables

	Test					
	TreeKernel		Univariate simes		Principal component F-test	
*15 omics variables*						
4-Partition						
2 Active groups	Group 1	Group 2	Group 1	Group 2	Group 1	Group 2
	**0.656**	0.348	0.003	0.017	**0.716**	**0.410**
1 Active group	0.848	0.141	**0.878**			
*30 omics variables*						
4-Partition						
2 Active groups	Group 1	Group 2	Group 1	Group 2	Group 1	Group 2
	**0.693**	**0.481**	0.004	0.006	0.530	0.398
1 Active group	**0.827**	0.024	0.680			
*45 omics variables*						
4-Partition						
2 Active groups	Group 1	Group 2	Group 1	Group 2	Group 1	Group 2
	**0.729**	**0.608**	0.005	0.007	0.426	0.374
1 Active group	**0.777**	0.024	0.546			

‘Group 1’ and ‘Group 2’ refer to the two partitions where the outcome was related to the simulated pathway

Bold cells indicate highest power in the simulation setting

Importantly, we see that TreeKernel is the only method with consistent power in the presence of multiple active partitions. The other methods have high power in detecting one partition but are often unable to detect the second. The estimated clusters from hierarchical clustering were also accurate. The average F1-scores ranges from 0.85 to 0.97 in all simulation settings. The hierarchical clustering did better with fewer clusters present, which also corresponded to the higher power we see in those simulation settings.

Table 4 shows the Type I error from 2000 simulations from multivariate normal distributions with 15, 30, and 45 omics variables using five components for clustering. All three methods maintain a Type I error rate close to the expected 0.05. In the 2-partition simulations the competing methods have a Type I error rate slightly closer to the expected 0.05, although this difference is negligible. See Additional file 1: Tables S4–S8 for the Type I error rates under the remaining simulation scenarios. We see that all methods maintain a Type I error rate reasonably close to the expected 0.05 in each simulation setting, although TreeKernel has a relatively low Type I error rate in larger simulated omics pathways. Again, we see this to be generally true in all simulation settings.

**Table 4:** Type I error from 2000 simulations with 15, 30, and 45 omics variables using five components with 1 continuous variable for clustering

	Test		
	TreeKernel	Univariate simes	Principal component F-test
*15 omics variables*			
4-partition	0.051	0.056	0.050
3-partition	0.051	0.041	0.053
2-Partition	0.057	0.050	0.047
*30 omics variables*			
4-partition	0.047	0.048	0.053
3-partition	0.047	0.046	0.048
2-Partition	0.057	0.040	0.058
*45 omics variables*			
4-partition	0.040	0.053	0.047
3-partition	0.040	0.054	0.052
2-Partition	0.049	0.052	0.052

### COPDGene analysis results

The clinical data partitions aligned almost perfectly with the subjects’ smoking status (current, former, never; Additional file 1: Table S9). Only 4 patients in the study were misclassified. There were only three metabolic pathways that were not significantly associated with the log FEV1/FVC ratio in at least one partition (smoking status). There were five that were significantly associated within each partition, but we will focus on the pathways where results differed among the partitions. Of the 29 pathways tested, there was one pathway significantly associated with the log FEV1/FVC ratio within the never-smoker group only, one pathway was significantly associated within the current-smokers group only, eleven were associated within the former-smokers group only, and six associated with 2 of the partitions. Of note, the $\beta$*-alanine metabolism* pathway was only associated with the never-smoker subgroup*,* The *tryptophan metabolism* pathway was only associated with the current-smoker subgroup, the pathways *glycine, serine, and threonine metabolism* and *neuroactive ligand-receptor interaction* were only associated with the former-smoker subgroup.

In the percent emphysema analysis, there were eight pathways that were not associated with any of the smoking subgroups. There were eighteen pathways that were only significantly associated with percent emphysema in the former-smoker subgroup, two associated with both the current- and former-smoker subgroups, and one associated with both the never- and former-smoker subgroups. Of note was the *arginine and proline metabolism* pathway which was associated in the current- and former-smoker subgroups. We will elaborate on the importance of these pathways in the current literature in the Discussion.

## Discussion

We have explored a method for interpretable interactions between high dimensional omics and clinical predictors with a continuous or binary clinical phenotype using kernel association tests and multivariate partitioning methods. Work has been done on interactions between and within multiple kernel spaces [10–12]. They still suffer from the ‘black box’ issue that many high-dimensional analysis techniques need to overcome. Interpreting and communicating interactions is often a challenge working within multidisciplinary teams, and these methods do not offer immediate interpretations of interactions. Our proposed method, TreeKernel, provides easily interpretable interactions between clinical spaces and kernelized spaces, which is an important piece to understanding biological processes. Our choice of hierarchical clustering may seem arbitrary, but we are in favor of having the addition information of the tree structure. When a deeper clustering structure exists, i.e., when the appropriate cut for clustering appears several nodes down the tree, there are benefits to using tree-structured *p*-value corrections [18].

Our simulations showed excellent power to detect multiple subgroups related to an outcome. Higher-dimensional kernel spaces may be interesting to explore, but our focus for this paper was on the analysis of smaller metabolic pathways. We note that TreeKernel’s power was slightly below FPC’s when the simulated pathways were small and there were many subgroups within the clinical data. However, we see higher power from TreeKernel in all other simulation settings. We also would like to stress the consistency of our method in the presence pathways related to the outcome within multiple subgroups. The power of TreeKernel was related to the accuracy of the estimated subgroups of the clinical data, so researchers should take the time to improve cluster quality when they can. However, improving clustering methods is not the focus of this paper, so we suggest hierarchical clustering with the standard relative inertia loss estimate for the number of clusters.

We were still able to detect pathways with multiple subgroup interactions in our analysis of the COPDGene data despite the low sample sizes. Moreover, our findings of these associations were consistent with prior research into COPD as well. The $\beta$*-alanine metabolism* pathway has been previously associated with COPD [40, 41]. The $\beta$*-alanine metabolism* and *Pantothenate and CoA biosynthesis* pathways have been previously associated with lung cancer patients and were significantly associated within our current-smokers [42]. The *tryptophan metabolism* pathway has been associated with acute exacerbations of COPD [43], and the *arginine metabolism* has documented upregulation in COPD patients [44].

In our analysis of the COPDGene data, we have clear grouping based on smoking status that aided with interpretation. Unsupervised clustering may not give such clear subgroups in other data sets. A factor analysis like the one we employ using the FactoMineR should give some insights into the variables driving the clusters. We posit that unexpected clinical grouping with clear interactions with a phenotype and a kernel space would make for excellent hypothesis generation. One should also be cautious about the size of the estimated subgroups, as smaller sample sizes can negatively impact kernel association tests. Different methods for creating embeddings of the clinical space, such as $kd$-trees, may also be beneficial depending on the setting. These will ensure larger sample sizes since the algorithm focuses on equal partitions, but this also mean the estimated clusters are not as driven by the clinical information.

Other kernel machines built to test for interaction, such as the garrote kernel [13], test for interactions between individual elements within the kernel. For our purposes, this would be equivalent to including a matrix of both the clinical and pathway variables, $A = \left[ {C,Z} \right]$, into the garrote kernel. However, users would not be able to know which elements of $A$ are interacting. Furthermore, ‘kernelizing’ clinical information would necessarily make all elements of $C$ continuous. Our approach allows for users to directly test of interactions between omics pathways and clinical subgroups, allowing for easier interpretations.

### Supplementary Information

**Additional file 1**. Supplemental provides simulation results not shown in the main text and the clustering results of the COPDGene clinical covariates.

## Data Availability

All code for simulations and analysis are available at https://github.com/Ghoshlab/TreeKernel. The metabolomics data set from the COPDGene Study can be found through PMID: 20214461 or DOI: 10.3109/15412550903499522.

## Abbreviations

COPD: Chronic obstructive pulmonary disease
FPC: F-test on principal components
