# Supplementary material for: TreeKernel: interpretable kernel machine tests for interactions between -omics and clinical predictors with applications to metabolomics and COPD phenotypes
Source: BMC Bioinformatics. 2023 Oct 25;24:398. doi: 10.1186/s12859-023-05459-x (PMC10601228; doi:10.1186/s12859-023-05459-x)
Supplement: Supplementary file 1 — Additional file 1. Supplemental provides simulation results not shown in the main text and the clustering results of the COPDGene clinical covariates. [file 12859_2023_5459_MOESM1_ESM.docx]

Supplementary Table 1: Estimated power from 2000 simulations from a multivariate normal distribution with 2, 3, or 4 partitions with 15, 30, and 45 omics variables. Tests used three principal components for clustering. Bold cells indicate the top performance within the simulation.

|  | Test | | | | | |
| --- | --- | --- | --- | --- | --- | --- |
| 15 Omics Variables | | | | | | |
|  | *TreeKernel* | | *Univariate Simes* | | *Principal Component F-test* | |
| **4-partition** |  | | | | | |
| 2 active groups | *Group 1* | *Group 2* | *Group 1* | *Group 2* | *Group 1* | *Group 2* |
|  | 0.555 | 0.199 | 0.012 | 0.022 | **0.673** | **0.334** |
| 1 active group | 0.752 | | 0.106 | | **0.823** | |
| **3-partition** |  | | | | | |
| 2 active groups | *Group 1* | *Group 2* | *Group 1* | *Group 2* | *Group 1* | *Group 2* |
|  | **0.889** | **0.574** | 0.021 | 0.023 | 0.833 | 0.018 |
| 1 active group | **0.976** | | 0.062 | | 0.918 | |
| **2-Partition** |  | | | | | |
| 1 active group | 0.955 | | 0.139 | | **0.962** | |
| 30 Omics Variables | | | | | | |
|  | *TreeKernel* | | *Univariate Simes* | | *Principal Component F-test* | |
| **4-partition** |  | | | | | |
| 2 active groups | *Group 1* | *Group 2* | *Group 1* | *Group 2* | *Group 1* | *Group 2* |
|  | **0.630** | **0.331** | 0.011 | 0.017 | 0.443 | 0.326 |
| 1 active group | **0.709** | | 0.088 | | 0.619 | |
| **3-partition** |  | | | | | |
| 2 active groups | *Group 1* | *Group 2* | *Group 1* | *Group 2* | *Group 1* | *Group 2* |
|  | **0.918** | **0.774** | 0.014 | 0.026 | 0.656 | 0.020 |
| 1 active group | **0.984** | | 0.068 | | 0.756 | |
| **2-Partition** |  | | | | | |
| 1 active group | **0.975** | | 0.138 | | 0.892 | |
| 45 Omics Variables | | | | | | |
|  | *TreeKernel* | | *Univariate Simes* | | *Principal Component F-test* | |
| **4-partition** |  | | | | | |
| 2 active groups | *Group 1* | *Group 2* | *Group 1* | *Group 2* | *Group 1* | *Group 2* |
|  | **0.650** | **0.413** | 0.013 | 0.016 | 0.359 | 0.318 |
| 1 active group | **0.653** | | 0.094 | | 0.500 | |
| **3-partition** |  | | | | | |
| 2 active groups | *Group 1* | *Group 2* | *Group 1* | *Group 2* | *Group 1* | *Group 2* |
|  | **0.910** | **0.891** | 0.014 | 0.025 | 0.551 | 0.016 |
| 1 active group | **0.963** | | 0.057 | | 0.659 | |
| **2-Partition** |  | | | | | |
| 1 active group | **0.958** | | 0.142 | | 0.799 | |

Supplementary Table 2: Estimated power from 2000 simulations with 15, 30, and 45 omics variables using 3 components for clustering with 1 categorical classifying variable. Bold cells indicate the top performance within the simulation.

|  | Test | | | | | |
| --- | --- | --- | --- | --- | --- | --- |
| 15 Omics Variables | | | | | | |
|  | *TreeKernel* | | *Univariate Simes* | | *Principal Component F-test* | |
| **4-partition** |  | | | | | |
| 2 active groups | *Group 1* | *Group 2* | *Group 1* | *Group 2* | *Group 1* | *Group 2* |
|  | 0.402 | 0.174 | 0.004 | 0.015 | **0.517** | **0.224** |
| 1 active group | 0.656 | | 0.123 | | **0.784** | |
| **3-partition** |  | | | | | |
| 2 active groups | *Group 1* | *Group 2* | *Group 1* | *Group 2* | *Group 1* | *Group 2* |
|  | **0.758** | **0.412** | 0.003 | 0.055 | 0.746 | 0.054 |
| 1 active group | **0.916** | | 0.150 | | 0.894 | |
| **2-Partition** |  | | | | | |
| 1 active group | **0.993** | | 0.098 | | 0.966 | |
| 30 Omics Variables | | | | | | |
|  | *TreeKernel* | | *Univariate Simes* | | *Principal Component F-test* | |
| **4-partition** |  | | | | | |
| 2 active groups | *Group 1* | *Group 2* | *Group 1* | *Group 2* | *Group 1* | *Group 2* |
|  | **0.441** | **0.258** | 0.005 | 0.011 | 0.355 | 0.246 |
| 1 active group | **0.607** | | 0.173 | | 0.562 | |
| **3-partition** |  | | | | | |
| 2 active groups | *Group 1* | *Group 2* | *Group 1* | *Group 2* | *Group 1* | *Group 2* |
|  | **0.820** | **0.632** | 0.004 | 0.064 | 0.571 | 0.054 |
| 1 active group | **0.929** | | 0.182 | | 0.731 | |
| **2-Partition** |  | | | | | |
| 1 active group | **0.992** | | 0.108 | | 0.845 | |
| 45 Omics Variables | | | | | | |
|  | *TreeKernel* | | *Univariate Simes* | | *Principal Component F-test* | |
| **4-partition** |  | | | | | |
| 2 active groups | *Group 1* | *Group 2* | *Group 1* | *Group 2* | *Group 1* | *Group 2* |
|  | **0.460** | **0.304** | 0.008 | 0.008 | 0.278 | 0.213 |
| 1 active group | **0.560** | | 0.188 | | 0.430 | |
| **3-partition** |  | | | | | |
| 2 active groups | *Group 1* | *Group 2* | *Group 1* | *Group 2* | *Group 1* | *Group 2* |
|  | **0.773** | **0.676** | 0.003 | 0.069 | 0.462 | 0.051 |
| 1 active group | **0.896** | | 0.194 | | 0.609 | |
| **2-Partition** |  | | | | | |
| 1 active group | **0.989** | | 0.108 | | 0.760 | |

Supplementary Table 3: Estimated power from 2000 simulations with 15, 30, and 45 omics variables using 3 components for clustering with 2 binary classifying variables. Bold cells indicate the top performance within the simulation.

|  | Test | | | | | |
| --- | --- | --- | --- | --- | --- | --- |
| 15 Omics Variables | | | | | | |
|  | *TreeKernel* | | *Univariate Simes* | | *Principal Component F-test* | |
| **4-partition** |  | | | | | |
| 2 active groups | *Group 1* | *Group 2* | *Group 1* | *Group 2* | *Group 1* | *Group 2* |
|  | 0.390 | 0.169 | 0.003 | 0.017 | **0.473** | **0.246** |
| 1 active group | 0.636 | | 0.141 | | **0.765** | |
| 30 Omics Variables | | | | | | |
|  | *TreeKernel* | | *Univariate Simes* | | *Principal Component F-test* | |
| **4-partition** |  | | | | | |
| 2 active groups | *Group 1* | *Group 2* | *Group 1* | *Group 2* | *Group 1* | *Group 2* |
|  | **0.466** | **0.255** | 0.005 | 0.015 | 0.361 | 0.239 |
| 1 active group | **0.629** | | 0.154 | | 0.563 | |
| 45 Omics Varibles | | | | | | |
|  | *TreeKernel* | | *Univariate Simes* | | *Principal Component F-test* | |
| **4-partition** |  | | | | | |
| 2 active groups | *Group 1* | *Group 2* | *Group 1* | *Group 2* | *Group 1* | *Group 2* |
|  | **0.465** | **0.332** | 0.005 | 0.016 | 0.267 | 0.211 |
| 1 active group | **0.568** | | 0.189 | | 0.452 | |

Supplementary Table 4: Type I error rates from 2000 simulations with 15, 30, and 45 omics variables using 3 components with 1 continuous variable for clustering.

|  | Test | | |
| --- | --- | --- | --- |
| 15 Omics Variables | | | |
|  | *TreeKernel* | *Univariate Simes* | *Principal Component F-test* |
| **4-partition** | 0.048 | 0.118 | 0.050 |
| **3-partition** | 0.048 | 0.081 | 0.053 |
| **2-Partition** | 0.042 | 0.217 | 0.047 |
| 30 Omics Variables | | | |
|  | *TreeKernel* | *Univariate Simes* | *Principal Component F-test* |
| **4-partition** | 0.057 | 0.130 | 0.053 |
| **3-partition** | 0.057 | 0.081 | 0.048 |
| **2-Partition** | 0.044 | 0.214 | 0.053 |
| 45 Omics Variables | | | |
|  | *TreeKernel* | *Univariate Simes* | *Principal Component F-test* |
| **4-partition** | 0.050 | 0.102 | 0.047 |
| **3-partition** | 0.050 | 0.077 | 0.052 |
| **2-Partition** | 0.049 | 0.219 | 0.052 |

Supplementary Table 5: Type I error rates from 2000 simulations with 15, 30, and 45 omics variables using 5 components with 1 categorical variable for clustering.

|  | Test | | |
| --- | --- | --- | --- |
| 15 Omics Variables | | | |
|  | *TreeKernel* | *Univariate Simes* | *Principal Component F-test* |
| **4-partition** | 0.044 | 0.030 | 0.060 |
| **3-partition** | 0.044 | 0.037 | 0.054 |
| **2-Partition** | 0.051 | 0.043 | 0.041 |
| 30 Omics Variables | | | |
|  | *TreeKernel* | *Univariate Simes* | *Principal Component F-test* |
| **4-partition** | 0.038 | 0.031 | 0.051 |
| **3-partition** | 0.038 | 0.039 | 0.046 |
| **2-Partition** | 0.048 | 0.038 | 0.051 |
| 45 Omics Variables | | | |
|  | *TreeKernel* | *Univariate Simes* | *Principal Component F-test* |
| **4-partition** | 0.059 | 0.031 | 0.052 |
| **3-partition** | 0.059 | 0.028 | 0.054 |
| **2-Partition** | 0.050 | 0.031 | 0.053 |

Supplementary Table 6: Type I error rates from 2000 simulations with 15, 30, and 45 omics variables using 3 components with 1 categorical variable for clustering.

|  | Test | | |
| --- | --- | --- | --- |
| 15 Omics Variables | | | |
|  | *TreeKernel* | *Univariate Simes* | *Principal Component F-test* |
| **4-partition** | 0.045 | 0.037 | 0.049 |
| **3-partition** | 0.045 | 0.055 | 0.040 |
| **2-Partition** | 0.051 | 0.043 | 0.042 |
| 30 Omics Variables | | | |
|  | *TreeKernel* | *Univariate Simes* | *Principal Component F-test* |
| **4-partition** | 0.047 | 0.037 | 0.049 |
| **3-partition** | 0.047 | 0.046 | 0.046 |
| **2-Partition** | 0.042 | 0.042 | 0.053 |
| 45 Omics Variables | | | |
|  | *TreeKernel* | *Univariate Simes* | *Principal Component F-test* |
| **4-partition** | 0.051 | 0.038 | 0.051 |
| **3-partition** | 0.051 | 0.043 | 0.053 |
| **2-Partition** | 0.050 | 0.035 | 0.051 |

Supplementary Table 7: Type I error rates from 2000 simulations with 15, 30, and 45 omics variables using 5 components with 2 binary variables for clustering.

|  | Test | | |
| --- | --- | --- | --- |
|  | *TreeKernel* | *Univariate Simes* | *Principal Component F-test* |
| 15 Omics Variables | 0.050 | 0.037 | 0.043 |
| 30 Omics Variables | 0.049 | 0.033 | 0.037 |
| 15 Omics Variables | 0.049 | 0.033 | 0.053 |

Supplementary Table 8: Type I error rates from 2000 simulations with 15, 30, and 45 omics variables using 3 components with 2 binary variables for clustering.

|  | Test | | |
| --- | --- | --- | --- |
|  | *TreeKernel* | *Univariate Simes* | *Principal Component F-test* |
| 15 Omics Variables | 0.046 | 0.037 | 0.045 |
| 30 Omics Variables | 0.052 | 0.039 | 0.040 |
| 45 Omics Variables | 0.053 | 0.032 | 0.057 |

Supplementary Table 9: Partitions of the COPDGene clinical covariates. These are obtained from hierarchical clustering from the first 5 left singular vectors from a factor analysis of the clinical covariates.

|  | Partition | | |
| --- | --- | --- | --- |
|  | *1* | *2* | *3* |
| **Current-Smoker** | 0 | 263 | 0 |
| **Former-Smoker** | 0 | 4 | 781 |
| **Never-Smoker** | 65 | 0 | 0 |
